# Effect of Synthetic Dietary Triglycerides: A Novel Research Paradigm for Nutrigenomics

**DOI:** 10.1371/journal.pone.0001681

**Published:** 2008-02-27

**Authors:** Linda M. Sanderson, Philip J. de Groot, Guido J. E. J. Hooiveld, Arjen Koppen, Eric Kalkhoven, Michael Müller, Sander Kersten

**Affiliations:** 1 Nutrigenomics Consortium, Top Institute (TI) Food and Nutrition, Wageningen, The Netherlands; 2 Nutrition, Metabolism and Genomics Group, Division of Human Nutrition, Wageningen University, Wageningen, The Netherlands; 3 Department of Metabolic and Endocrine Diseases, Universitair Medisch Centrum (UMC) Utrecht, Utrecht, The Netherlands; Texas Tech University Health Sciences Center, United States of America

## Abstract

**Background:**

The effect of dietary fats on human health and disease are likely mediated by changes in gene expression. Several transcription factors have been shown to respond to fatty acids, including SREBP-1c, NF-κB, RXRs, LXRs, FXR, HNF4α, and PPARs. However, it is unclear to what extent these transcription factors play a role in gene regulation by dietary fatty acids *in vivo*.

**Methodology/Principal Findings:**

Here, we take advantage of a unique experimental design using synthetic triglycerides composed of one single fatty acid in combination with gene expression profiling to examine the effects of various individual dietary fatty acids on hepatic gene expression in mice. We observed that the number of significantly changed genes and the fold-induction of genes increased with increasing fatty acid chain length and degree of unsaturation. Importantly, almost every single gene regulated by dietary unsaturated fatty acids remained unaltered in mice lacking PPARα. In addition, the majority of genes regulated by unsaturated fatty acids, especially docosahexaenoic acid, were also regulated by the specific PPARα agonist WY14643. Excellent agreement was found between the effects of unsaturated fatty acids on mouse liver versus cultured rat hepatoma cells. Interestingly, using Nuclear Receptor PamChip® Arrays, fatty acid- and WY14643-induced interactions between PPARα and coregulators were found to be highly similar, although several PPARα-coactivator interactions specific for WY14643 were identified.

**Conclusions/Significance:**

We conclude that the effects of dietary unsaturated fatty acids on hepatic gene expression are almost entirely mediated by PPARα and mimic those of synthetic PPARα agonists in terms of regulation of target genes and molecular mechanism. Use of synthetic dietary triglycerides may provide a novel paradigm for nutrigenomics research.

## Introduction

Dietary fatty acids have multiple functions in the human body. They are an important energy source, form an essential part of the phospholipid bilayer of membranes, and function as precursors to several signaling molecules, such as the eicosanoids. A huge body of literature collected in the past few decades provides compelling evidence that changes in the dietary fatty acid composition can profoundly influence health and disease. For example, it is well established that replacing dietary saturated fatty acids with n-6 mono- and polyunsaturated leads to a decrease in plasma concentration of low density lipoprotein, which is a well-known risk factor for atherosclerosis [1]. Likewise, increased consumption of n-3 fatty acids, especially eicosapentaenoic acid and docosahexaenoic acid present in fish oil, is associated with decreased plasma triglyceride concentrations [2], may prevent against cardiac arrhythmias [3], and improves visual acuity in preterm infants [4]. Numerous molecular mechanisms may underlie the effects of dietary fatty acids on parameters of health. While historically the main focus was on changes in plasma membrane fluidity as a result of changes in phospholipid composition, the discovery of nuclear receptors has progressively shifted the emphasis to regulation of gene expression.

The superfamily of nuclear receptors encompasses a related but diverse set of transcription factors that share a number of structural and functional features [5]. They consist of a central DNA-binding domain that directs the receptor to specific DNA sequences within a gene promoter, and a ligand-binding domain, which can accommodate a variety of different compounds. Roughly, nuclear receptors can be divided into three main groups: the endocrine receptors that bind steroid hormones, the adopted orphan receptors that bind dietary lipids, and the orphan receptors, for which no ligand exists or still has to be identified [6]. The adopted orphan receptors share a common mode of action that involves heterodimerization with the nuclear Retinoid X Receptor (RXR). Binding of ligands to the receptor leads to recruitment of co-activators and dissociation of co-repressors, resulting in chromatin remodeling followed by initiation of DNA transcription. Adopted orphan receptors mainly function as lipid sensors by altering the rate of transcription of specific genes in response to changes in lipid concentration [6]. These lipids include oxysterols, bile acids, and fat soluble vitamins. In addition, many adopted orphan receptors have been shown to bind fatty acids and alter transcription in response to changes in fatty acid concentration and/or composition, including RXR, Peroxisome-Proliferator Activated Receptors (PPARα, β/δ and γ), Hepatic Nuclear Factor 4α (HNF-4α), Liver X Receptor (LXR) α and β, and Farnesoid X Receptor [7], [8]. Other receptors that mediate the effects of dietary fatty acids on gene expression include the Sterol Regulatory Element Binding Protein 1, and the Nuclear factor kappaB [7]. However, the relative contribution of all these receptors to fatty acid-dependent gene regulation in vivo remains completely unclear.

Here, we take advantage of a unique experimental design using synthetic triglycerides composed of one single fatty acid in combination with gene expression profiling to examine the effects of individual dietary fatty acids on hepatic gene expression in mice. By conducting these experiments in wild-type and PPARα −/− mice, we were able to explore the specific contribution of PPARα. We conclude that the effects of dietary unsaturated fatty acids on hepatic gene expression are almost exclusively mediated by PPARα and mimic those of synthetic PPARα agonists in terms of target genes regulation and molecular mechanism.

## Results

**Figure 1:**
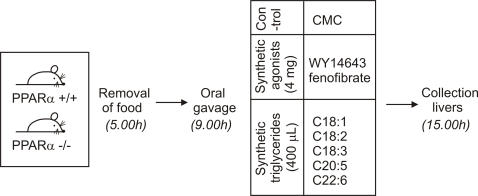
Schematic representation of dietary intervention. Wild-type and PPARα −/− mice fasted for 4 hours were given a single oral dose of different synthetic triglycerides composed of one single unsaturated fatty acid (400 µl), or one of the PPARα agonists WY14643 or fenofibrate (4 mg). After 6h, the livers were used for gene expression profiling using Affymetrix Mouse Genome 430 2.0 Microarrays (∼45000 probesets) on biological replicates. CMC = carboxymethyl cellulose.

Mice that were fasted for 4 hours were given a single oral dose (400 µl) of synthetic triglycerides (TGs) consisting of one single fatty acid, followed by collection of tissues 6 hours thereafter (Figure 1). A parallel treatment in mice lacking PPARα was performed to enable estimation of the importance of PPARα in gene regulation by dietary fatty acids. The fatty acids studied were oleic acid (C18:1), linoleic acid (C18:2), linolenic acid (C18:3), eicosapentaenoic acid (C20:5), and docosahexaenoic acid (C22:6). No saturated fatty acids were included because triglycerides composed of common dietary saturated fatty acids are solid at room temperature and could not be administered orally. The 6-hour time point was chosen because in an independent oral fat load experiment, plasma triglyceride (TG) levels peaked 2 hours after the fat load and almost returned back to baseline after 6 hours (Figure S1A), indicating that at that point most of the fat bolus has been cleared from the blood and taken up by the tissues. Indeed, we observed that 6 hours after oral dosing plasma TG levels had almost returned to baseline (Figure S1B), and were similar in WT and PPARα −/− mice, suggesting no major differences in plasma TG kinetics between the various fatty acids and between WT and PPARα −/−. Also, no major differences in the rate of intestinal TG absorption were observed between WT and PPARα −/− mice (Figure S1C). Finally, while as expected liver TG levels were higher in the PPARα −/− mice compared to WT mice, in the WT mice liver TG levels were similar between the various fatty acids (Figure S1D). These data argue against major differences in metabolic processing of dietary fat between WT and PPARα −/− mice and between different dietary fatty acids.

The focus of the present study is on liver since we observed that, when expressed per gram organ weight, the liver and heart take up most of the fatty acids present in TG-rich lipoproteins (Figure S1E). A future publication will address the effect of dietary fatty acids on gene expression and the involvement of PPARs in heart.

### PPARα-dependent gene regulation by dietary unsaturated fatty acids

Expression profiling was carried out on individual mouse livers. Use of Affymetrix Mouse Genome 430 2.0 Arrays (whole mouse genome array), which contain more than 45000 probesets corresponding to over 34000 genes, allows for a genome-wide analysis of the number of significantly changed genes in the various treatment groups. After inter-quartile range (IQR) filtering, 11463 probesets (equivalent to 7231 genes) were left for analysis. A regularized t-test was performed to analyze changes in gene expression between the control and oral triglyceride group. The regularized t-test statistic has the same interpretation as an ordinary t-test statistic, except that the standard errors have been moderated across genes, i.e. shrunk to a common value, using a Bayesian model [9]. A probeset was found to be significantly changed after treatment if P<0.01. All microarray results have been deposited into the Gene Expression Omnibus (http://www.ncbi.nlm.nih.gov/projects/geo/) and can be accessed online under series number GSE8396. Quantitative real-time PCR was carried out on ∼30 genes in order to confirm the results from the microarray, and the results were found to be in close agreement with the microarray data (Figure S2).

**Figure 2:**
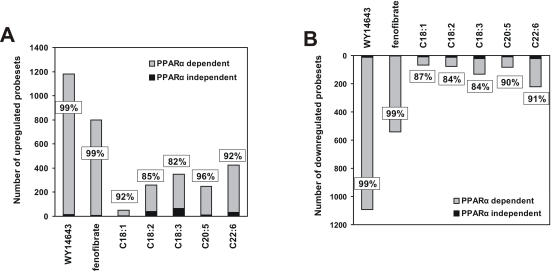
PPARα-dependent regulation of gene expression by dietary unsaturated fatty acids. Bars show number of up- (upper panel) and downregulated (lower panel) probesets in the different treatment groups. The number of probesets regulated by unsaturated fatty acids in a PPARα-dependent manner (light bars, not changed in the PPARα −/− mouse), or PPARα-independent manner (dark bars, changed in wild-type and PPARα −/− mice) are shown, with percentage PPARα dependence indicated. Probesets were considered statistically significantly regulated if P<0.01.

The highest number of statistically significantly changed genes was found after treatment with C22:6 (519, P<0.01), followed by, in turn, C18:3 (400), C18:2 (287), C20:5 (280) and C18:1 (114) (Figure 2 and table S1). These numbers are relatively low in comparison with the synthetic PPARα agonists WY14643 (1674) and fenofibrate (1005). The data indicate that of all fatty acids studied, C22:6 is the most potent activator of gene expression.

Regulation of gene expression by dietary fatty acids or synthetic agonists was defined as PPARα-dependent when expression was statistically significantly up- or downregulated in WT but not PPARα −/− mice. As expected, gene regulation by WY14643 and fenofibrate in WT mice was almost completely abolished in PPARα −/− mice. Surprisingly, a similar though slightly less extreme picture was observed for dietary unsaturated fatty acids. Indeed, the far majority of genes regulated by dietary unsaturated fatty acids in WT mice did not show regulation in PPARα −/− mice, indicating PPARα-dependent regulation. This was highest for C20:5 (94.6%), followed by C22:6 (93.1%), C18:1 (88.6%), C18:2 (87.1%) and C18:3 (84.0%) (Figure 2 and table S1). Similar numbers were obtained for up- and downregulation of gene expression. The few genes that were up- or downregulated by dietary unsaturated fatty acids independently of PPARα included *Lpin2* and *Srebp-1*, respectively. Together, these data suggest that the (short term) effects of dietary unsaturated fatty acids on hepatic gene expression are almost exclusively mediated by PPARα.

### Overlap in gene regulation between dietary unsaturated fatty acids and WY14643

**Figure 3:**
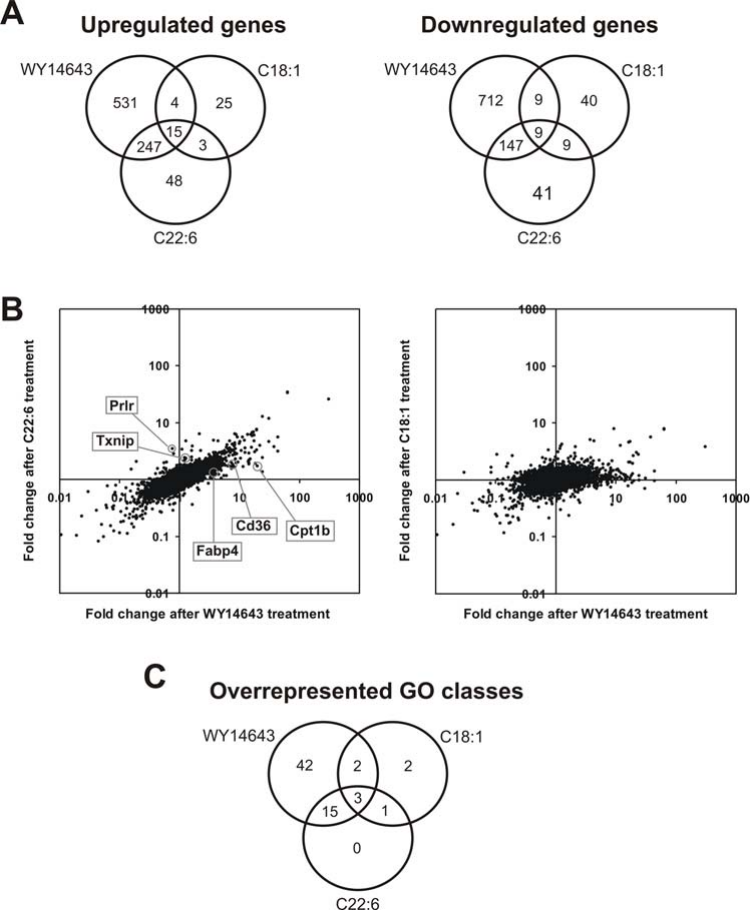
Similarities between two dietary unsaturated fatty acids and the synthetic PPARα agonist WY14643. (A) Venn diagrams showing the overlap in up- (left panel) and downregulated (right panel) genes after treatment with WY14643, C22:6 and C18:1. Genes were considered statistically significantly regulated if P<0.01. (B) Scatter plots demonstrating similarities in gene regulation between C22:6 and WY14643. Graphs show fold change in gene expression after treatment with WY14643 compared to C22:6 and C18:1. Genes that are upregulated disproportionally strongly by WY14643 (*Cd36*, *Fabp4* (*aP2*), and *Cpt1b*), or by C22:6 (*Prlr* and *Txnip*) are marked. In constructing the scatter plots, all probesets left after IQR-filtering were used. (C) Overlap in overrepresented Gene Ontology classes between C22:6, C18:1, and WY14643, based on a functional class score (FCS) method. The GO class unique to C22:6 and C18:1 is GO:0016070 (RNA metabolism), whereas the GO classes unique to C18:1 are GO:0007409 (axonogenesis) and GO:0016072 (rRNA metabolism).

To further explore the role of PPARα in regulation of gene expression by dietary unsaturated fatty acids, the overlap in gene regulation between fatty acids and WY14643, which specifically targets PPARα, was studied. Remarkably, C22:6 showed a huge overlap in gene regulation with WY14643 (Figure 3A). Quantitatively, 84% of genes upregulated and 76% of genes downregulated by C22:6 (P<0.01) were also regulated by WY14643 (average 80.5%), suggesting that C22:6 impacts mainly PPARα target genes. Much less overlap was observed between C18:1 and WY14643 (average 32.4%), suggesting that gene regulation by C18:1 may be less dependent on PPARα, or alternatively the existence of PPARα target genes specifically regulated by C18:1 (Figure 3A). An intermediate degree of overlap was observed between WY14643 and the other fatty acids studied (Table S2).

To further compare the effects of WY14643 and C22:6 on gene expression, for all probesets left after IQR-filtering the fold-changes in expression in response to WY14643 and C22:6 were plotted against each other, with each probeset represented by a single dot (Figure 3B). The vast majority of probesets ended up in the lower left or upper right compartments, indicating that genes up- or downregulated by WY14643 were also up- or downregulated by C22:6, respectively, thus confirming the overlap in gene regulation between C22:6 and WY14643. Additionally, the positioning of the dots around a straight line with slope <1 shows that the relative magnitude of gene induction by C22:6 related to WY14643 was remarkably constant across all probesets. Thus, compared to WY14643, C22:6 behaves as an almost equally specific, yet less potent PPARα agonist. Nevertheless, several genes could be identified that were upregulated disproportionally strongly by WY14643 including *Cd36*, *Fabp4* (*aP2*), and *Cpt1b*, or by C22:6 including *Prlr* and *Txnip* (Figure 3B). A much more scattered picture was observed for the comparison between WY14643 and C18:1, indicating that these compounds have much less in common in terms of gene regulation. Again, the other fatty acids gave an intermediate picture (data not shown).

An alternative approach to study similarities in gene regulation is via determining the overlap in Gene Ontology (GO) classes overrepresented in the respective treatment groups (Table S3). P-values derived from t-test for all ∼45000 probesets on the microarray were used for the GO-based functional clustering. The comparisons were made between the control group and each treatment group in wild-type mice. Out of a total of 19 GO classes overrepresented after C22:6 treatment, only one class (GO:0016070, RNA metabolism) was not shared between C22:6 and WY14643 (Figure 3C). Interestingly, this GO class was shared between C22:6 and C18:1 suggesting it may be specifically regulated by dietary unsaturated fatty acids and not WY14643. The remainder of fatty acids studied, except for perhaps C18:1, similarly showed a high degree of overlap with WY14643 (Table S4), thereby corroborating the very large resemblance in gene regulation between WY14643 and the dietary fatty acids studied. Overall, these data support the dominant role of PPARα in gene regulation by dietary unsaturated fat.

### Hierarchy between dietary unsaturated fatty acids

**Figure 4:**
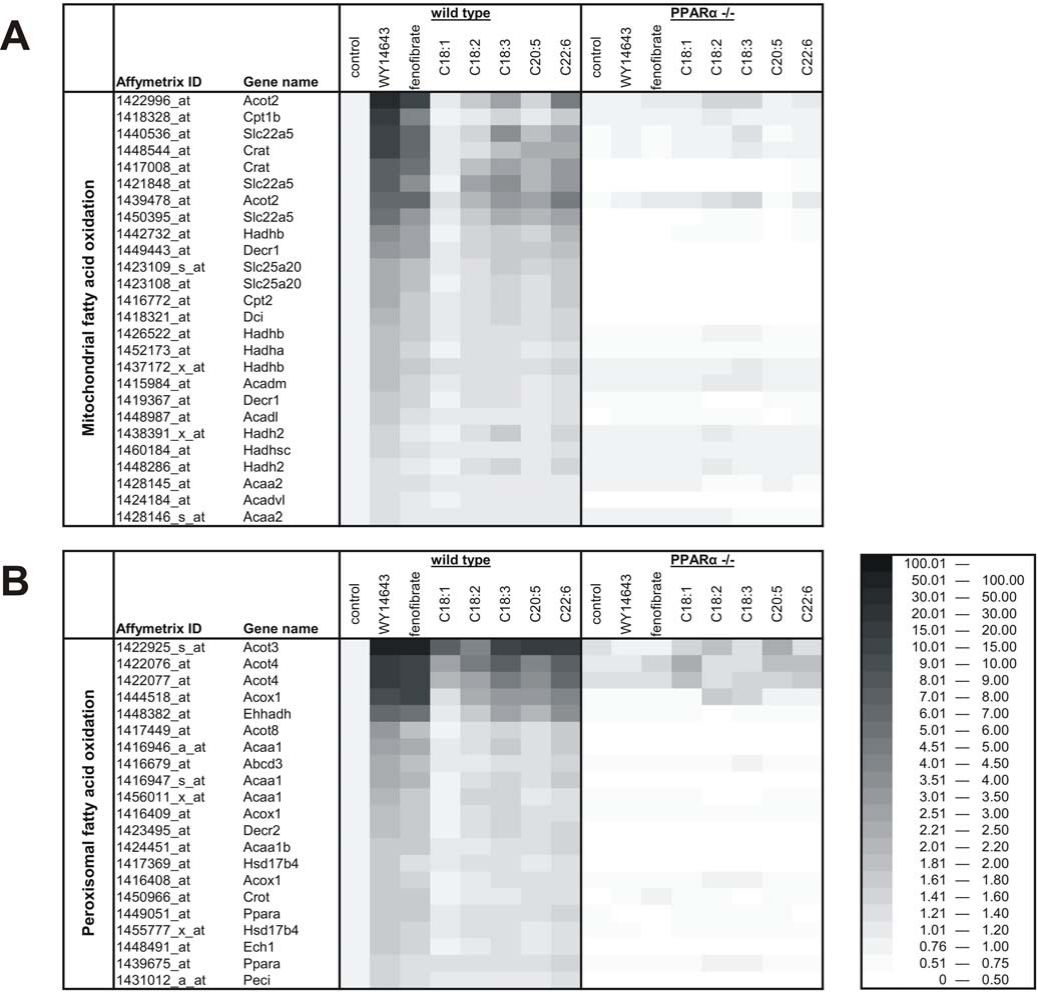
Differential induction of genes involved in fatty acid oxidation between various dietary fatty acids. (A) Genes involved in mitochondrial fatty acid oxidation. (B) Genes involved in peroxisomal fatty acid oxidation. The heatmaps were generated directly from the microarray data, using for each probeset the mean signal from 4–5 biological replicates. The grayscale represents fold-induction relative to wild-type control, which was set at 1. Only probesets showing significant (P<0.01) upregulation by WY14643 were included in the analysis. A list of probesets belonging to the functional class of mitochondrial fatty acid oxidation can be found at http://nutrigene.4t.com/microarray/ppar2007.

Of all fatty acids studied, the number of significantly changed genes was highest for C22:6, followed by C18:3. The number was about equal for C20:5 and C18:2, while much fewer genes were changed after C18:1 treatment. Since the dietary fatty acids regulated gene expression principally via PPARα, the data are indicative of a hierarchy in *in vivo* PPARα-activating potency between dietary unsaturated fatty acids. Direct evidence for this notion came from comparison of fold-changes in expression of PPARα target genes between the various fatty acid treatments. Genes involved in two major PPARα-regulated pathways were examined: mitochondrial fatty acid oxidation and peroxisomal fatty acid oxidation (Figure 4). These functional classes were created in house for various pathways within lipid metabolism and were specifically designed for Affymetrix GeneChip analysis (available at http://nutrigene.4t.com/microarray/ppar2007). By visualizing the changes in gene expression in the form of a heatmap, a clear hierarchy in PPARα-activating potency can be observed between the various treatments, which can be expressed as WY>feno>22:6>20:5 = 18:3>18:2>18:1.

Since a direct comparison between synthetic agonists and dietary fatty acids is complicated by differences in dosage (4 mg vs. 400 µl), further comparisons were made between fatty acids only. For all probesets shown in the heatmaps as well as probesets belonging to the lipogenesis pathway we estimated the relative induction by each fatty acid expressed as a percentage of induction by C22:6. The median for all probesets within a functional class was calculated for each treatment group (Figure S3, bar). These data indicate that C22:6 is the most potent activator of PPARα-dependent gene regulation in mouse liver, while C18:1 is the least active.

**Figure 5:**
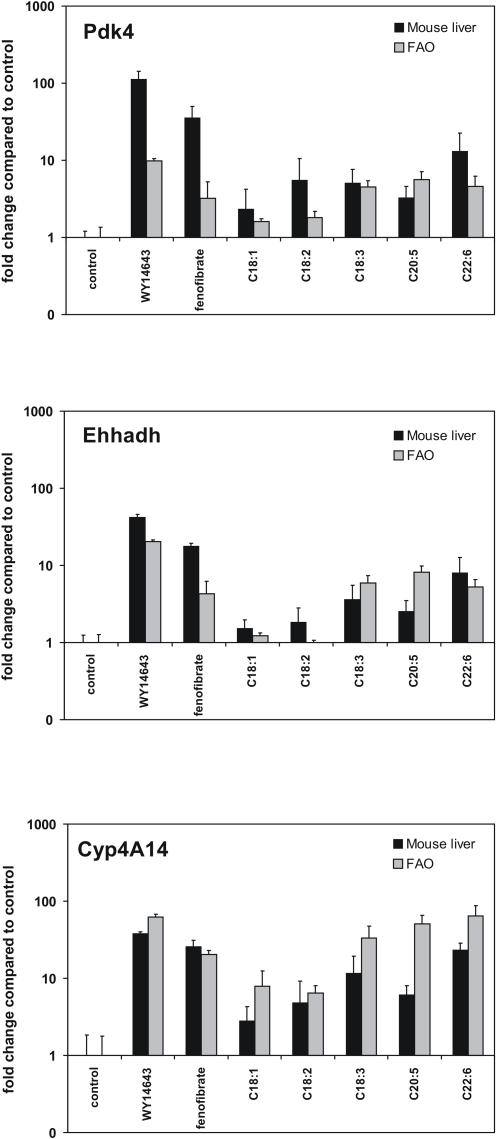
Close agreement between fatty acid-dependent gene regulation *in vivo* and *in vitro*. mRNA expression of three genes (*Pdk4*, *Ehhadh* and *Cyp4A14*) was determined in mouse liver and in rat hepatoma FAO cells using quantitative real-time PCR. Results are shown as fold-change compared to control group. Error bars represent SD.

To examine whether the difference in *in vivo* PPARα-activating potency between the dietary fatty acids could be reproduced *in vitro*, cultured rat FAO hepatoma cells were treated with various unsaturated fatty acids. It was observed that the pattern of regulation of PPARα targets *Pdk4*, *Ehhadh* and *Cyp4A14* by unsaturated fatty acids was highly similar between the FAO cells and intact mouse liver (Figure 5). These data provide additional evidence that differences in metabolic processing of fatty acids are unlikely to explain differential fold-induction of genes between dietary fatty acids observed *in vivo*. Rather, they indicate an intrinsic difference in PPARα-activating potency between dietary unsaturated fatty acids, which is supported by published *in vitro* data.

**Figure 6:**
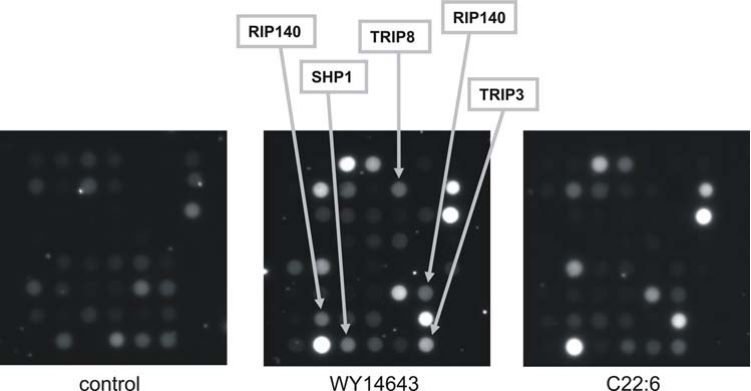
Cofactor recruitment assay with WY14643 and C22:6. The Nuclear Receptor PamChip® assay was used to measure the interaction between PPARα and immobilized peptides corresponding to specific coregulator-nuclear receptor binding regions. Measurements were performed in the presence of control (EtOH), WY14643 (5 µM) or C22:6 (100 µM). Arrows point to those co-activators selectively recruited by WY14643 but not C22:6. All images were taken after 100 msec exposure time.

While in terms of target gene regulation dietary unsaturated fatty acids thus generally mimic the effect of the synthetic PPARα agonist WY14643 except for being less potent, it is unclear whether these different compounds activate PPARα and stimulate transcription of target genes via the exact same mechanism. To explore this issue we used Nuclear Receptor PamChip® Arrays to identify differences in coregulator recruitment between WY14643 and C22:6. In this system the interaction between nuclear receptors and immobilized peptides corresponding to specific coregulator-nuclear receptor binding regions is studied. Both C22:6 and WY14643 promoted the interaction between PPARα and numerous coregulator peptides. Interestingly, no PPARα-coregulator interactions unique to C22:6 could be identified. However, at least 4 interactions, representing the coregulator proteins TRIP3, TRIP8, RIP140, and the nuclear receptor SHP1, seemed to be elicited specifically by WY14643 (Figure 6). No differences in PPARα-coregulator interaction patterns could be observed between the various fatty acids studied (data not shown).

## Discussion

Dietary fats have numerous effects on human health. Current dietary guidelines strongly discourage consumption of saturated and trans fatty acids, whereas consumption of unsaturated fatty acids, especially n-3 fatty acids present in fish oil, is promoted [10]–[12]. It is believed that dietary fatty acids mainly influence biological processes by altering DNA transcription. In the present paper, using a unique dietary intervention protocol consisting of a single dose of synthetic triglycerides composed of a single fatty acid, we show that in mouse liver PPARα dominates gene regulation by dietary unsaturated fat. Furthermore, we demonstrate that dietary PUFAs, especially docosahexaenoic acid, are the most potent activators of PPARα *in vivo*. These latter data align well with *in vitro* experiments showing that in general PUFAs are more potent PPARα ligands compared to mono- and saturated fatty acids, although the results may depend somewhat on the method used [13]–[19].

It can be argued that our data and conclusions may be biased due to possible differential absorption and metabolic processing between the various fatty acids and between WT and PPARα −/− mice. Unfortunately, the unavailability of radioactive TG besides triolein makes it impossible to get complete and comparative information on the kinetic behavior of the various fatty acids used. However, several lines of evidence argue against major differences in kinetic behavior between the fatty acids: 1) it has been previously demonstrated that hepatic uptake of fatty acids from chylomicron remnants is unaffected by the fatty acid composition. [20]; 2) at the moment of sacrifice, plasma TG levels were highly similar for the various fatty acid groups; 3) fatty acid treatment *in vivo* and *in vitro* revealed a similar hierarchy in PPARα-activating potency between the fatty acids and in both analyses C22:6 emerged as the most potent PPARα agonist.

In addition, no major differences in the kinetics of dietary fat metabolism are expected between WT and PPARα −/− mice as: 1) WT and PPARα −/− mice show similar rates of intestinal TG absorption; 2) at the moment of sacrifice, plasma TG levels were highly similar between WT and PPARα −/− mice; 3) while synthetic PPARα agonists are known to stimulate plasma TG clearance [21], no evidence is available that points to differences in plasma TG clearance and tissue fatty acid uptake between WT and PPARα −/− mice; 4) genes that are upregulated by fatty acids in a PPARα-independent manner were induced to the same extent in WT and PPARα −/− mice (data not shown), suggesting that the dietary fatty acids were taken up at the same rate in liver of WT and PPARα −/− mice.

While PPARα activity is known to respond to changes in dietary fat content and composition [22]–[24], the large dominance of PPARα in fatty acid-dependent gene regulation in liver is surprising given that the activity of numerous transcription factors can be modulated by fatty acids, including SREBP-1, HNF4α, LXRs, FXR, RXRs, NF-κB, as well as PPARβ/δ and PPARγ [25]–[37]. For several of these proteins, including RXRs and HNF4α, physical binding by fatty acids or fatty acyl-CoAs has been demonstrated [30]–[32], [38]–[42]. RXR forms a permissive heterodimer with PPARα and accordingly it may be theorized that transcriptional activation of PPAR target genes by fatty acids may occur via their binding to either the PPARα and/or RXR moiety. The loss of fatty acid-dependent gene regulation in PPARα −/− mice, the very large overlap in gene regulation between unsaturated fatty acids and WY14643, and the less potent binding of fatty acids to RXR relative to PPARα strongly suggest a dominant role for PPARα in gene regulation by unsaturated fatty acids [28], [31], [43], [44]. However, an additional role for RXR is hard to exclude as the effects of RXR activation seem to occur primarily via PPARα [45]. It remains to be investigated to what extent the dominant role of PPARα in gene regulation by unsaturated fatty acids extends to tissues other than liver. Likely, the relative role of other transcription factors is related to their relative expression in a particular tissue.

Although it is clear that gene regulation by unsaturated fatty acids is highly dependent on PPARα, genes that are regulated in a PPARα-dependent manner do not necessarily represent direct PPARα targets. Some regulation is also expected to occur indirectly via activation of other transcription factors that are under direct control of PPARα. Analysis of the microarray data showed very little changes in the expression of other nuclear receptors in response to the intervention with the exception of CAR, which was upregulated, and RXRα and AhR, which were downregulated, although not necessarily in all treatments. The nuclear receptor CAR was recently identified as a PPARα target [46], suggesting that some genes may be regulated by PPARα and fatty acids via CAR. Secondary gene regulation was likely kept to a minimum by harvesting the livers only six hours after the oral gavage. It should also be noted that none of the putative fatty acid responsive transcription factors were significantly decreased in PPARα −/− mice, suggesting that their transcriptional regulatory function is not intrinsically suppressed in PPARα −/− mice.

Our study shows a clear hierarchy between unsaturated dietary fatty acids in terms of number of significantly changed genes and fold-induction of genes, with especially C22:6 behaving as a highly potent inducer of PPARα-dependent gene expression. The difference in *in vivo* PPARα-activating potency between the dietary fatty acids was reproduced *in vitro* and thus suggest an intrinsic difference in PPARα-activating potency between dietary unsaturated fatty acids, which is supported by *in vitro* receptor binding and transactivation studies and thus likely reflects differences in binding affinity for PPARα [19], [25]–[29]. Even though C18:2 was not the most potent inducer of gene expression, one could speculate that it likely represents the quantitatively most important dietary activator of PPARα, as the average intake of C18:2 is much higher than that of C18:3, C20:5 and C22:6.

In recent years, the concept of Selective PPAR Modulators (SPPARM) has emerged by analogy to Selective Estrogen Receptor Modulators (SERM). According to this concept, different PPAR agonists would induce differential gene expression based on selective receptor-coregulator interactions. While recent evidence supports the concept of selective PPARγ modulation [47]–[49], only limited data are available on PPARα [50]. The design of our study allowed us to explore the concept of SPPARM in the comparison between unsaturated fatty acids and synthetic agonists. We hypothesized that fatty acids and synthetic PPARα agonists, while both activating PPARα, may induce differential gene expression patterns possibly via selective receptor-coregulator interactions.

In our analysis we found that almost every gene significantly up- or downregulated by C22:6 was also significantly up- or downregulated by WY14643, respectively. Clearly, the reverse was not true, illustrating that WY14643 is a more potent PPARα agonist than C22:6. Importantly, the scatter plot indicated that across all probesets the relative induction of gene expression by C22:6 when related to WY14643 was remarkably constant, suggesting that C22:6 behaves as a less potent, yet almost equally specific PPARα agonist. Nevertheless, several genes could be identified that were upregulated disproportionally strongly by WY14643 including *Cd36*, *Fabp4* (*aP2*), and *Cpt1b*, or by C22:6 including *Prlr* and *Txnip* (Figure 3B). Thus, differences in gene regulation between C22:6 and WY14643 could not entirely be accounted for by the lesser potency of C22:6. Interestingly, using the Nuclear Receptor PamChip® assay, at least 4 interactions, representing the coregulator proteins TRIP3, TRIP8, RIP140, and the nuclear receptor SHP1, seemed to be stimulated specifically by WY14643. However, no PPARα-coregulator interactions could be identified that were stimulated specifically by C22:6 and not WY14643.

Overall, similar observations were made for the other fatty acids studied, although compared to C22:6 they were less potent and/or less specific activators of PPARα, especially C18:1. The data indicate that in general dietary PUFAs mimic the effect of WY14643 on hepatic gene expression in terms of regulation of target genes and molecular mechanism, including coregulator interactions. In addition to being a more potent PPARα agonist in comparison with unsaturated fatty acids, WY14643 dysproportionally induces expression of specific genes, which may be mediated via interactions with specific coactivator proteins including RIP140. Thus, our data underscore the concept of selective PPARα modulation when comparing WY14643 with endogenous PPARα agonists, e.g. PUFAs.

Currently, one major drawback when performing microarray analyses on data derived from dietary intervention studies is the lack of proper statistical tools. The statistical methods developed to cope with the huge amount of data derived from microarray analyses work sufficiently well for stronger interventions, such as drug studies. When dealing with nutrition, however, changes in gene expression are often weak although no less important. Multiple testing methods normally used in microarray analyses to correct for false positives include FDR (false discovery rate) and Q-value [51]–[53]. These methods are usually too restrictive for nutritional intervention, however, and will result in a loss of important results, as became apparent in the present study. Use of Q-value instead of P-value resulted in loss of a considerable amount of important information (data not shown). Numerous quantitative real-time PCR reactions have been carried out on the livers from this study supporting the use of the P-value.

In conclusion, dietary unsaturated fatty acids, especially docosahexaenoic acid and other PUFAs, acutely influence gene expression in mouse liver which, despite the presence of numerous other putative fatty acid-dependent transcription factors, is almost entirely mediated by PPARα. Consequently, dietary PUFAs largely mimic the effect of synthetic PPARα agonists on hepatic gene expression, both in terms of regulation of specific target genes and molecular mechanism including coregulator interactions, although compared to WY14643 and fenofibrate they are clearly less potent PPARα agonists. Our analysis underscores the power of a (nutri)genomics approach to investigate the potential molecular mechanisms underlying the effect of specific dietary components on (biomarkers of) health.

## Materials and Methods

### Materials

WY14643 was purchased from ChemSyn Laboratories (Lenexa, KS, USA). Triolein was from Fluka (Zwijndrecht, The Netherlands). Trilinolein, trilinolenin, tridocosahexaenoin and trieicosapentaenoin were from Nu-Chek-Prep, Inc. (Elysian, MN, USA). Fetal bovine serum and penicillin/streptomycin were from Cambrex Bioscience (Seraing, Belgium). SYBR Green was purchased from Eurogentec (Seraing, Belgium). All other chemicals were from Sigma (Zwijndrecht, The Netherlands).

### Animals

Male pure-bred SV129 and PPARα −/− mice (2–6 months of age) on a SV129 background were used. Two weeks before start of the experiment, the animals were switched to a run-in diet consisting of a modified AIN76A diet (corn oil was replaced by olive oil) (Research Diet Services, Wijk bij Duurstede, The Netherlands). Starting at 5 a.m. the animals were fasted for 4 hours followed by an intragastric gavage of 400 µl synthetic triglyceride (triolein, trilinolein, trilinolenin, trieicosapentaenoin or tridocosahexaenoin) (Figure 1). WY14643 and fenofibrate were given as 10 mg/ml suspension in 0.5% carboxymethyl cellulose, which also served as control treatment (400 µl). Four to five mice per group were used, adding up to 78 mice in total. 6 hours after gavage, mice were anaesthetized with a mixture of isofluorane (1.5%), nitrous oxide (70%) and oxygen (30%). Blood was collected by orbital puncture, after which the mice were sacrificed by cervical dislocation. Livers were removed, snap-frozen in liquid nitrogen and stored at −80°C until further analysis. For RNA analyses, tissue from the same part of the liver lobe was used.

The animal studies were approved by the Local Committee for Care and Use of Laboratory Animals at Wageningen University.

### Lipid absorption and tissue distribution

Measurement of intestinal lipid absorption was carried out exactly as previously described [54]. For the lipid loading test WT mice were fasted for 4 hours followed by administration of 400 µl olive oil via intragastric gavage. Blood was collected by tail bleeding every 2 hours for plasma TG measurement. Tissue uptake of [^3^H]-labeled TG packaged into VLDL-like emulsion particles was measured as previously described [55]. The data shown reflect percentage of bolus radioactivity taken up after 30 minutes by a specific tissue expressed per gram tissue.

### Triglycerides

Plasma and liver triglycerides were measured with a commercially available kit from Instruchemie (Delfzijl, The Netherlands). Livers were weighed and homogenized in a buffer (pH 7.5) containing 250 mM sucrose, 1 mM EDTA and 10 mM Tris, with a final tissue concentration of 5%. 2 µl of plasma or liver homogenate was used to determine TG.

### RNA isolation and qRT-PCR

Total liver RNA was isolated with TRIzol reagent (Invitrogen, Breda, The Netherlands) according to manufacturer's instructions. A NanoDrop ND-1000 spectrophotometer (Isogen, Maarssen, The Netherlands) was used to determine RNA concentrations. 1 µg of total RNA was reverse transcribed using iScript (Bio-Rad, Veenendaal, The Netherlands). cDNA was amplified on a Bio-Rad MyIQ or iCycler PCR machine using Platinum Taq DNA polymerase (Invitrogen, Breda, The Netherlands). PCR primer sequences were taken from the PrimerBank [56] and ordered from Eurogentec (Seraing, Belgium). Sequences of the primers used are available upon request.

### Affymetrix microarray

Total RNA from mouse liver was extracted with TRIzol reagent, and purified and DNAse treated using the SV Total RNA Isolation System (Promega, Leiden, The Netherlands). RNA quality was assessed on an Agilent 2100 bioanalyzer (Agilent Technologies, Amsterdam, the Netherlands) with 6000 Nano Chips using the Eukaryote Total RNA Nano assay. RNA was judged as suitable for array hybridization only if samples showed intact bands corresponding to the 18S and 28S rRNA subunits, displayed no chromosomal peaks or RNA degradation products, and had a RIN (RNA integrity number) above 8.0. Five micrograms of RNA were used for one cycle cRNA synthesis (Affymetrix, Santa Clara, CA). Hybridization, washing and scanning of Affymetrix Mouse Genome 430 2.0 arrays was carried out according to standard Affymetrix protocols.

Packages from the Bioconductor project were used for analyzing the scanned Affymetrix arrays [57]. Arrays were normalized using quartile normalization, and expression estimates were compiled using GC-RMA applying the empirical Bayes approach [58]. A non-specific filtering step was applied to remove probesets with low variation, as they provide no discriminating power [59]. Only those probesets were included that had an inter-quartile range (IQR) across the samples of at least 0.25 on the log2-scale. Differentially expressed probesets were identified using linear models, applying moderated t-statistics that implement empirical Bayes regularization of standard errors [9].

Comparisons were made between wild-type treated and untreated (control) and also between PPARα −/− treated and untreated animals. Probesets that presented a P-value <0.01 were considered to be significantly changed by treatment. If a probeset was significantly changed in the wild-type but not the PPARα −/− mouse, it was considered to be PPARα-dependent (also probesets that were significantly changed in the PPARα −/− mouse, but had a fold-change <1.5 of the fold-change in the wild-type mouse were included in this category).

Functional analysis of the array data was performed by a method based on overrepresentation of Gene Ontology (GO) terms, where the functional class score (FCS) method was used [60]–[62].

### Cell culture

Rat hepatoma FAO cells were grown in DMEM containing 10% (vol/vol) fetal bovine serum, 100 U/ml penicillin and 100 µg/ml streptomycin. Cells were incubated with albumin-bound fatty acids (100 µM) dissolved in ethanol or synthetic PPARα ligands dissolved in DMSO (5 µM WY14643, 50 µM fenofibrate). Incubation continued for 24 hours and was followed by RNA isolation and qRT-PCR.

### Cofactor recruitment assay

Nuclear Receptor PamChip® Arrays (PamGene, s'Hertogenbosch, The Netherlands) were used according to the manufacturer's instructions. Upon binding a ligand, PPARα undergoes a conformational change which promotes the formation of a cofactor binding pocket, subsequently allowing interaction with the so-called LxxLL motif within some coregulators. The PamChip® arrays consist of 48 peptides encompassing the LxxLL motifs of 19 different coregulator proteins ([63], Koppen et al. 2007. Micro Array assay for Real-time analysis of Coregulator-Nuclear receptor Interaction. Manuscript submitted.) Briefly, the arrays were incubated with glutathione-S-transferase (GST)-tagged PPARα-LBD (Invitrogen, Breda, The Netherlands) in the presence and absence of ligand. Quantification of interaction between PPARα and coregulators was made using Alexa488-conjugated anti-GST rabbit polyclonal antibody (Invitrogen). As ligands, either a negative control (EtOH), the synthetic PPARα agonist WY14643 or one of the fatty acids C18:1, C18:2, C18:3, C20:5 or C22:6 were used.

## Supporting Information

Figure S1 Metabolic processing of dietary triglycerides. (A) WT mice were given an oral fat load of 400 µl olive oil via intragastric gavage. TG levels were measured in plasma collected via the tail vein at the indicated time points. Errors bars represent SEM (n = 11). (B) Plasma TG of WT and PPARα −/− mice sacrificed 6 hours after intragastric gavage with synthetic triglycerides, WY14643, or fenofibrate. Error bars represent SD (n = 4–5 per group). (C) Intestinal triglyceride absorption rate was determined in 5h fasted WT and PPARα −/− mice by measuring the appearance of [^3^H] in plasma after intragastric gavage with 7uCi glycerol-tri[^3^H]oleate mixed with olive oil (200 µl). Immediately before the gavage, mice received an intraorbital injection of tyloxapol (Triton WR1339) dissolved in saline at 500 mg/kg bodyweight. Blood was sampled via the tail vein at the indicated time points for measurement of ^3^H-activity. Error bars represent SEM. (D) Liver TG of WT and PPARα −/− mice sacrificed 6 hours after intragastric gavage with synthetic triglycerides, WY14643, or fenofibrate. Error bars represent SD (n = 4–5 per group). (D) Tissue uptake of radiolabeled VLDL-like emulsion particles. VLDL-like particles labeled with glycerol tri[^3^H]oleate were injected into anesthetized mice. After 30 minutes, mice were euthanized and tissues collected for measurement of ^3^H-activity. Error bars represent SEM (n = 4).
(1.62 MB TIF)

Figure S2 Close agreement between microarray and quantitative real-time PCR data. mRNA expression of several genes was measured by quantitative real-time PCR to confirm the results from microarray. Results are shown as fold-change compared to wild-type control. Error bars represent SD.
(2.12 MB TIF)

Figure S3 Differential induction of genes involved in lipid metabolism between dietary unsaturated fatty acids. For each probeset, the induction of expression by each fatty acid was expressed as a percentage relative to C22:6 (100%), using the mean signal from 4–5 biological replicates. Each dot represents one probeset. The horizontal bars represent the median percentage of induction relative to C22:6 calculated separately for each pathway and fatty acid. Only probesets showing significant (P<0.01) upregulation by WY14643 were included in the analysis. A list of probesets belonging to the three functional classes can be found at http://nutrigene.4t.com/microarray/ppar2007.
(0.81 MB TIF)

Table S1 Total number as well as PPARα dependent up- and downregulated probesets and corresponding genes for each treatment group (P<0.01).
(0.07 MB DOC)

Table S2 Overlap in gene regulation between dietary unsaturated fatty acids and WY14643. Genes were considered statistically significantly regulated if P<0.01.
(0.04 MB DOC)

Table S3 Overrepresented GO classes in each treatment group based on analysis with Functional Class Score method, FDR<0.0001.
(0.39 MB DOC)

Table S4 Overlap in overrepresented Gene Ontology classes between dietary unsaturated fatty acids and fenofibrate and WY14643 based on analysis with Functional Class Score method, FDR<0.0001.
(0.04 MB DOC)
