# Supplementary figures and images for: Effect of Synthetic Dietary Triglycerides: A Novel Research Paradigm for Nutrigenomics
Source: PLoS One. 2008 Feb 27;3(2):e1681. doi: 10.1371/journal.pone.0001681 (PMC2244803; doi:10.1371/journal.pone.0001681)

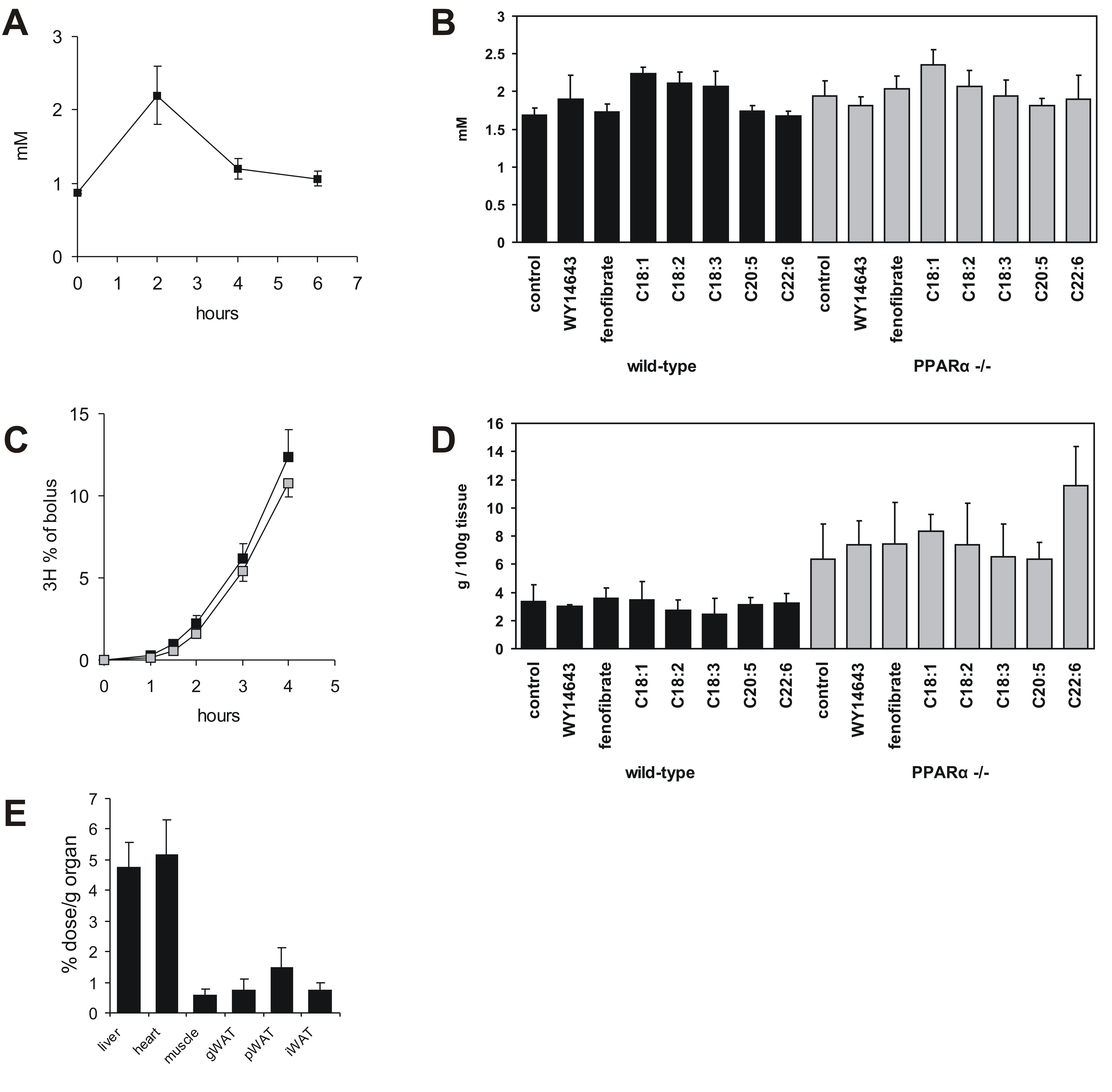

Supplement: Figure S1 — Metabolic processing of dietary triglycerides. (A) WT mice were given an oral fat load of 400 µl olive oil via intragastric gavage. TG levels were measured in plasma collected via the tail vein at the indicated time points. Errors bars represent SEM (n = 11). (B) Plasma TG of WT and PPARα −/− mice sacrificed 6 hours after intragastric gavage with synthetic triglycerides, WY14643, or fenofibrate. Error bars represent SD (n = 4–5 per group). (C) Intestinal triglyceride absorption rate was determined in 5h fasted WT and PPARα −/− mice by measuring the appearance of [3H] in plasma after intragastric gavage with 7uCi glycerol-tri[3H]oleate mixed with olive oil (200 µl). Immediately before the gavage, mice received an intraorbital injection of tyloxapol (Triton WR1339) dissolved in saline at 500 mg/kg bodyweight. Blood was sampled via the tail vein at the indicated time points for measurement of 3H-activity. Error bars represent SEM. (D) Liver TG of WT and PPARα −/− mice sacrificed 6 hours after intragastric gavage with synthetic triglycerides, WY14643, or fenofibrate. Error bars represent SD (n = 4–5 per group). (D) Tissue uptake of radiolabeled VLDL-like emulsion particles. VLDL-like particles labeled with glycerol tri[3H]oleate were injected into anesthetized mice. After 30 minutes, mice were euthanized and tissues collected for measurement of 3H-activity. Error bars represent SEM (n = 4). (1.62 MB TIF) [file pone.0001681.s001.tif]

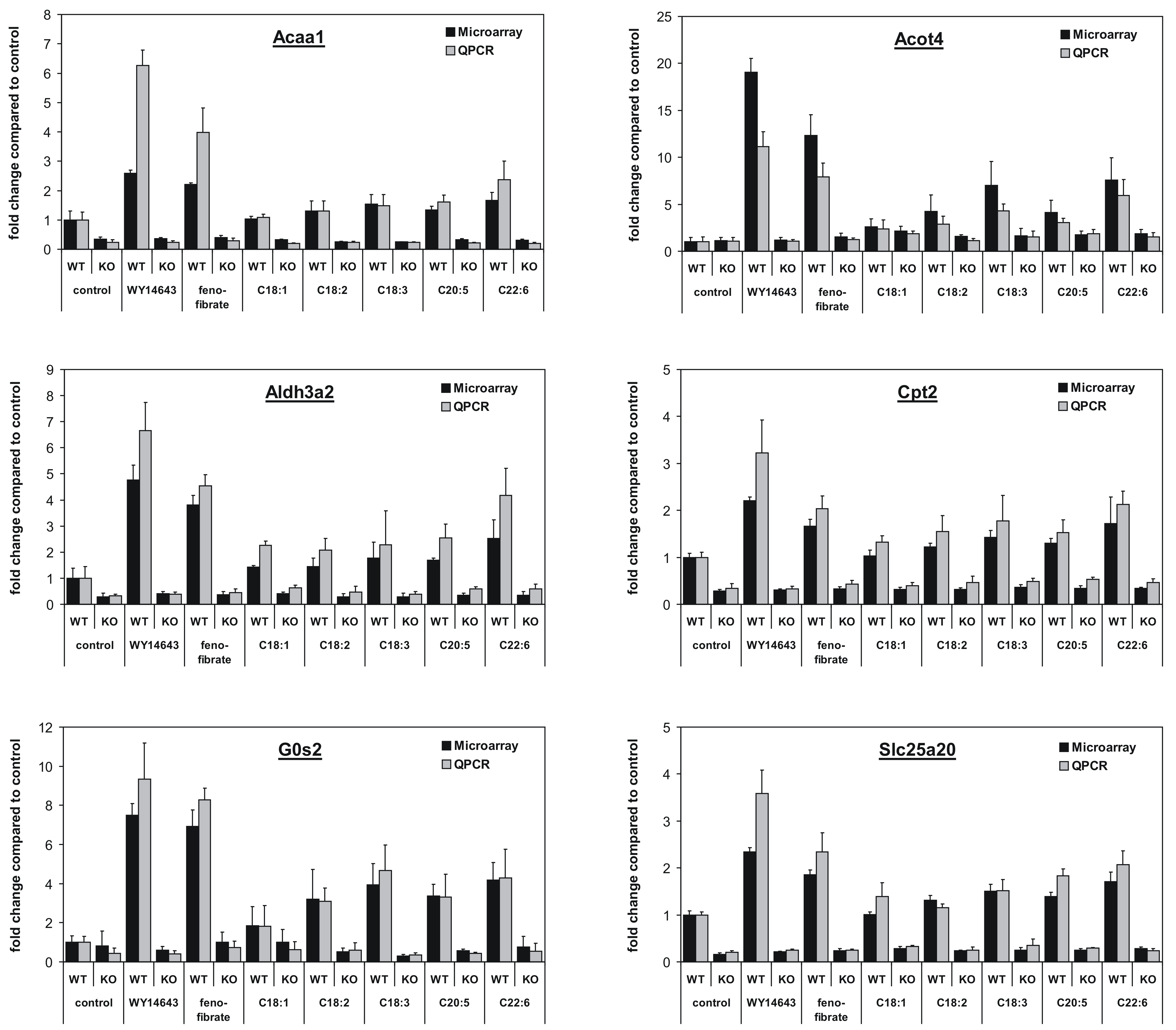

Supplement: Figure S2 — Close agreement between microarray and quantitative real-time PCR data. mRNA expression of several genes was measured by quantitative real-time PCR to confirm the results from microarray. Results are shown as fold-change compared to wild-type control. Error bars represent SD. (2.12 MB TIF) [file pone.0001681.s002.tif]

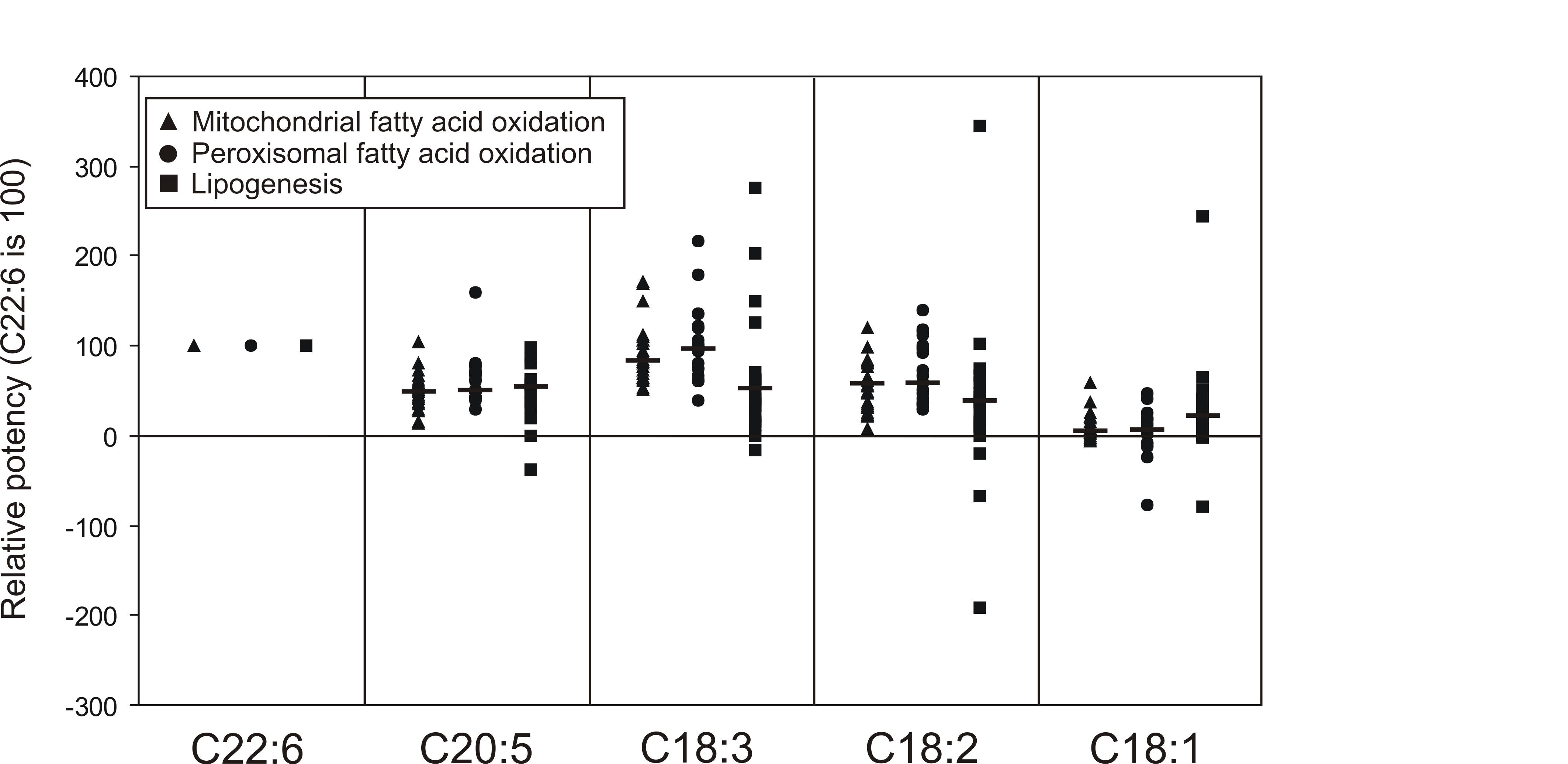

Supplement: Figure S3 — Differential induction of genes involved in lipid metabolism between dietary unsaturated fatty acids. For each probeset, the induction of expression by each fatty acid was expressed as a percentage relative to C22:6 (100%), using the mean signal from 4–5 biological replicates. Each dot represents one probeset. The horizontal bars represent the median percentage of induction relative to C22:6 calculated separately for each pathway and fatty acid. Only probesets showing significant (P<0.01) upregulation by WY14643 were included in the analysis. A list of probesets belonging to the three functional classes can be found at http://nutrigene.4t.com/microarray/ppar2007. (0.81 MB TIF) [file pone.0001681.s003.tif]
