# Supplementary material for: Effect of Synthetic Dietary Triglycerides: A Novel Research Paradigm for Nutrigenomics
Source: PLoS One. 2008 Feb 27;3(2):e1681. doi: 10.1371/journal.pone.0001681 (PMC2244803; doi:10.1371/journal.pone.0001681)
Supplement: Table S1 — Total number as well as PPARα dependent up- and downregulated probesets and corresponding genes for each treatment group (P<0.01). (0.07 MB DOC) [file pone.0001681.s004.doc]

|  | **Probesets** | | | **Genes** | | |  |
| --- | --- | --- | --- | --- | --- | --- | --- |
|  | **Total number** | **PPARα dependent** | **% dependent** | **Total number** | **PPARα dependent** | **% dependent** |  |
| WY14643 | 1180 | 1165 | 98.7% | 797 | 789 | 99.0% | **Upregulated** |
| fenofibrate | 800 | 794 | 99.3% | 549 | 544 | 99.1% |
| C18:1 | 51 | 47 | 92.2% | 47 | 44 | 93.6% |
| C18:2 | 260 | 221 | 85.0% | 212 | 186 | 87.7% |
| C18:3 | 349 | 285 | 81.7% | 283 | 237 | 83.7% |
| C20:5 | 249 | 238 | 95.6% | 201 | 194 | 96.5% |
| C22:6 | 425 | 393 | 92.5% | 313 | 296 | 94.6% |
| WY14643 | 1092 | 1081 | 99.0% | 877 | 867 | 98.9% | **Downregulated** |
| fenofibrate | 540 | 537 | 99.4% | 456 | 454 | 99.6% |
| C18:1 | 69 | 60 | 87.0% | 67 | 57 | 85.1% |
| C18:2 | 77 | 65 | 84.4% | 75 | 64 | 85.3% |
| C18:3 | 133 | 112 | 84.2% | 117 | 99 | 84.6% |
| C20:5 | 84 | 76 | 90.5% | 79 | 71 | 89.9% |
| C22:6 | 221 | 201 | 91.0% | 206 | 187 | 90.8% |
| WY14643 | 2272 | 2246 | 98.9% | 1674 | 1656 | 98.9% | **Up- and downregulated** |
| fenofibrate | 1340 | 1331 | 99.3% | 1005 | 998 | 99.3% |
| C18:1 | 120 | 107 | 89.2% | 114 | 101 | 88.6% |
| C18:2 | 337 | 286 | 84.9% | 287 | 250 | 87.1% |
| C18:3 | 482 | 397 | 82.4% | 400 | 336 | 84.0% |
| C20:5 | 333 | 314 | 94.3% | 280 | 265 | 94.6% |
| C22:6 | 646 | 594 | 92.0% | 519 | 483 | 93.1% |
