# Supplementary material for: Effect of Synthetic Dietary Triglycerides: A Novel Research Paradigm for Nutrigenomics
Source: PLoS One. 2008 Feb 27;3(2):e1681. doi: 10.1371/journal.pone.0001681 (PMC2244803; doi:10.1371/journal.pone.0001681)
Supplement: Table S2 — Overlap in gene regulation between dietary unsaturated fatty acids and WY14643. Genes were considered statistically significantly regulated if P<0.01. (0.04 MB DOC) [file pone.0001681.s005.doc]

| **Treatment** | **Number of changed genes** | **Number of changed genes overlapping with WY14643** | **% changed genes overlapping with WY14643** |
| --- | --- | --- | --- |
| WY14643 | 1674 |  |  |
| fenofibrate | 1005 | 875 | 87.1% |
| C18:1 | 114 | 37 | 32.4% |
| C18:2 | 287 | 142 | 49.5% |
| C18:3 | 400 | 244 | 61.0% |
| C20:5 | 280 | 220 | 78.6% |
| C22:6 | 519 | 418 | 80.5% |
