# Supplementary material for: Effect of Synthetic Dietary Triglycerides: A Novel Research Paradigm for Nutrigenomics
Source: PLoS One. 2008 Feb 27;3(2):e1681. doi: 10.1371/journal.pone.0001681 (PMC2244803; doi:10.1371/journal.pone.0001681)
Supplement: Table S3 — Overrepresented GO classes in each treatment group based on analysis with Functional Class Score method, FDR<0.0001. (0.39 MB DOC) [file pone.0001681.s006.doc]

|  | **GO ID** | **GO Class** | **Probes in class** | **Genes in class** | **Raw score** | **FDR** |  |
| --- | --- | --- | --- | --- | --- | --- | --- |
| 1 | GO:0006637 | acyl-CoA metabolism | 24 | 14 | 4.56 | 3.16E-11 | **WY14643** |
| 2 | GO:0001676 | long-chain fatty acid metabolism | 11 | 9 | 3.60 | 8.87E-05 |
| 3 | GO:0007031 | peroxisome organization and biogenesis | 31 | 16 | 3.08 | 2.89E-11 |
| 4 | GO:0006732 | coenzyme metabolism | 63 | 35 | 2.87 | 2.41E-11 |
| 5 | GO:0006635 | fatty acid beta-oxidation | 21 | 14 | 2.79 | 8.15E-05 |
| 6 | GO:0019395 | fatty acid oxidation | 38 | 21 | 2.53 | 2.73E-11 |
| 7 | GO:0006090 | pyruvate metabolism | 42 | 20 | 2.46 | 3.26E-11 |
| 8 | GO:0019752 | carboxylic acid metabolism | 215 | 115 | 2.36 | 4.40E-11 |
| 9 | GO:0019319 | hexose biosynthesis | 43 | 21 | 2.27 | 4.04E-11 |
| 10 | GO:0008654 | phospholipid biosynthesis | 92 | 34 | 2.19 | 3.37E-11 |
| 11 | GO:0006469 | negative regulation of protein kinase activity | 70 | 26 | 2.12 | 7.22E-11 |
| 12 | GO:0009408 | response to heat | 51 | 25 | 1.94 | 2.53E-10 |
| 13 | GO:0046467 | membrane lipid biosynthesis | 92 | 34 | 1.86 | 9.19E-11 |
| 14 | GO:0006869 | lipid transport | 118 | 60 | 1.85 | 2.25E-11 |
| 15 | GO:0009266 | response to temperature stimulus | 68 | 38 | 1.77 | 5.95E-11 |
| 16 | GO:0006725 | aromatic compound metabolism | 60 | 34 | 1.71 | 2.35E-11 |
| 17 | GO:0006644 | phospholipid metabolism | 116 | 45 | 1.68 | 4.21E-11 |
| 18 | GO:0008203 | cholesterol metabolism | 122 | 55 | 1.64 | 5.62E-11 |
| 19 | GO:0006986 | response to unfolded protein | 138 | 64 | 1.63 | 1.12E-10 |
| 20 | GO:0006333 | chromatin assembly or disassembly | 110 | 37 | 1.59 | 8.72E-05 |
| 21 | GO:0051789 | response to protein stimulus | 123 | 56 | 1.57 | 1.01E-09 |
| 22 | GO:0045859 | regulation of protein kinase activity | 83 | 33 | 1.57 | 8.29E-05 |
| 23 | GO:0009607 | response to biotic stimulus | 145 | 73 | 1.57 | 2.53E-11 |
| 24 | GO:0007050 | cell cycle arrest | 122 | 49 | 1.55 | 2.11E-11 |
| 25 | GO:0012502 | induction of programmed cell death | 182 | 81 | 1.53 | 2.66E-11 |
| 26 | GO:0008202 | steroid metabolism | 210 | 104 | 1.53 | 2.06E-11 |
| 27 | GO:0008610 | lipid biosynthesis | 230 | 108 | 1.52 | 1.44E-10 |
| 28 | GO:0016125 | sterol metabolism | 114 | 52 | 1.51 | 1.94E-11 |
| 29 | GO:0006694 | steroid biosynthesis | 119 | 56 | 1.45 | 5.32E-11 |
| 30 | GO:0043065 | positive regulation of apoptosis | 283 | 117 | 1.44 | 2.47E-11 |
| 31 | GO:0043067 | regulation of programmed cell death | 238 | 107 | 1.43 | 8.43E-11 |
| 32 | GO:0045786 | negative regulation of progression through cell cycle | 273 | 108 | 1.41 | 2.15E-11 |
| 33 | GO:0035023 | regulation of Rho protein signal transduction | 145 | 63 | 1.41 | 3.06E-11 |
| 34 | GO:0045087 | innate immune response | 83 | 56 | 1.39 | 1.26E-10 |
| 35 | GO:0006260 | DNA replication | 282 | 108 | 1.39 | 2.97E-11 |
| 36 | GO:0006917 | induction of apoptosis | 241 | 106 | 1.38 | 6.32E-11 |
| 37 | GO:0006006 | glucose metabolism | 86 | 47 | 1.38 | 8.43E-05 |
| 38 | GO:0007507 | heart development | 255 | 102 | 1.36 | 5.06E-11 |
| 39 | GO:0001525 | angiogenesis | 209 | 92 | 1.30 | 1.91E-11 |
| 40 | GO:0007243 | protein kinase cascade | 278 | 109 | 1.30 | 3.49E-11 |
| 41 | GO:0008284 | positive regulation of cell proliferation | 209 | 94 | 1.29 | 3.61E-11 |
| 42 | GO:0007266 | Rho protein signal transduction | 207 | 87 | 1.29 | 1.84E-11 |
| 43 | GO:0006916 | anti-apoptosis | 189 | 75 | 1.28 | 5.06E-10 |
| 44 | GO:0016568 | chromatin modification | 284 | 107 | 1.27 | 1.01E-10 |
| 45 | GO:0043066 | negative regulation of apoptosis | 284 | 115 | 1.26 | 3.74E-11 |
| 46 | GO:0042060 | wound healing | 106 | 62 | 1.26 | 8.57E-05 |
| 47 | GO:0045934 | negative regulation of nucleobase, nucleoside, nucleotide and nucleic acid metabolism | 179 | 70 | 1.26 | 2.30E-11 |
| 48 | GO:0016481 | negative regulation of transcription | 274 | 109 | 1.25 | 1.87E-11 |
| 49 | GO:0006814 | sodium ion transport | 198 | 105 | 1.24 | 2.02E-11 |
| 50 | GO:0000165 | MAPKKK cascade | 214 | 85 | 1.23 | 1.98E-11 |
| 51 | GO:0007067 | mitosis | 268 | 114 | 1.21 | 6.74E-11 |
| 52 | GO:0048514 | blood vessel morphogenesis | 236 | 98 | 1.19 | 4.81E-11 |
| 53 | GO:0051242 | positive regulation of cellular physiological process | 201 | 91 | 1.18 | 4.60E-11 |
| 54 | GO:0040008 | regulation of growth | 215 | 89 | 1.18 | 2.81E-11 |
| 55 | GO:0019538 | protein metabolism | 215 | 101 | 1.17 | 1.81E-11 |
| 56 | GO:0030036 | actin cytoskeleton organization and biogenesis | 299 | 112 | 1.17 | 1.69E-10 |
| 57 | GO:0001558 | regulation of cell growth | 228 | 94 | 1.16 | 2.20E-11 |
| 58 | GO:0051056 | regulation of small GTPase mediated signal transduction | 195 | 86 | 1.16 | 2.02E-10 |
| 59 | GO:0006325 | establishment and/or maintenance of chromatin architecture | 299 | 112 | 1.15 | 7.78E-11 |
| 60 | GO:0000087 | M phase of mitotic cell cycle | 239 | 104 | 1.15 | 3.89E-11 |
| 61 | GO:0006092 | main pathways of carbohydrate metabolism | 206 | 99 | 1.15 | 3.37E-10 |
| 62 | GO:0045045 | secretory pathway | 288 | 115 | 1.10 | 2.59E-11 |

|  | **GO ID** | **GO Class** | **Probes in class** | **Genes in class** | **Raw score** | **FDR** |  |
| --- | --- | --- | --- | --- | --- | --- | --- |
| 1 | GO:0006637 | acyl-CoA metabolism | 24 | 14 | 4.66 | 4.04E-11 | **fenofibrate** |
| 2 | GO:0001676 | long-chain fatty acid metabolism | 11 | 9 | 2.87 | 1.26E-10 |
| 3 | GO:0006732 | coenzyme metabolism | 63 | 35 | 2.78 | 3.06E-11 |
| 4 | GO:0006084 | acetyl-CoA metabolism | 28 | 9 | 2.58 | 9.54E-05 |
| 5 | GO:0007031 | peroxisome organization and biogenesis | 31 | 16 | 2.46 | 3.61E-11 |
| 6 | GO:0006635 | fatty acid beta-oxidation | 21 | 14 | 2.31 | 2.30E-11 |
| 7 | GO:0009725 | response to hormone stimulus | 35 | 13 | 2.20 | 3.37E-10 |
| 8 | GO:0006090 | pyruvate metabolism | 42 | 20 | 2.12 | 4.21E-11 |
| 9 | GO:0019395 | fatty acid oxidation | 38 | 21 | 1.98 | 3.49E-11 |
| 10 | GO:0019752 | carboxylic acid metabolism | 215 | 115 | 1.90 | 6.74E-11 |
| 11 | GO:0019319 | hexose biosynthesis | 43 | 21 | 1.85 | 5.95E-11 |
| 12 | GO:0006519 | amino acid and derivative metabolism | 57 | 27 | 1.76 | 3.89E-11 |
| 13 | GO:0006695 | cholesterol biosynthesis | 50 | 22 | 1.72 | 5.06E-10 |
| 14 | GO:0008654 | phospholipid biosynthesis | 92 | 34 | 1.68 | 4.60E-11 |
| 15 | GO:0016126 | sterol biosynthesis | 59 | 25 | 1.66 | 1.12E-10 |
| 16 | GO:0009308 | amine metabolism | 65 | 30 | 1.65 | 2.81E-11 |
| 17 | GO:0008203 | cholesterol metabolism | 122 | 55 | 1.45 | 8.43E-11 |
| 18 | GO:0046467 | membrane lipid biosynthesis | 92 | 34 | 1.39 | 1.69E-10 |
| 19 | GO:0006725 | aromatic compound metabolism | 60 | 34 | 1.37 | 2.97E-11 |
| 20 | GO:0006869 | lipid transport | 118 | 60 | 1.27 | 2.89E-11 |
| 21 | GO:0007050 | cell cycle arrest | 122 | 49 | 1.27 | 2.66E-11 |
| 22 | GO:0016125 | sterol metabolism | 114 | 52 | 1.26 | 2.47E-11 |
| 23 | GO:0043283 | biopolymer metabolism | 88 | 39 | 1.26 | 9.72E-05 |
| 24 | GO:0008610 | lipid biosynthesis | 230 | 108 | 1.26 | 2.53E-10 |
| 25 | GO:0006644 | phospholipid metabolism | 116 | 45 | 1.25 | 6.32E-11 |
| 26 | GO:0006694 | steroid biosynthesis | 119 | 56 | 1.19 | 7.78E-11 |
| 27 | GO:0006986 | response to unfolded protein | 138 | 64 | 1.17 | 2.02E-10 |
| 28 | GO:0007243 | protein kinase cascade | 278 | 109 | 1.15 | 4.81E-11 |
| 29 | GO:0043067 | regulation of programmed cell death | 238 | 107 | 1.15 | 1.44E-10 |
| 30 | GO:0045786 | negative regulation of progression through cell cycle | 273 | 108 | 1.14 | 2.73E-11 |
| 31 | GO:0008202 | steroid metabolism | 210 | 104 | 1.12 | 2.59E-11 |
| 32 | GO:0012502 | induction of programmed cell death | 182 | 81 | 1.06 | 3.37E-11 |
| 33 | GO:0007507 | heart development | 255 | 102 | 1.06 | 7.22E-11 |
| 34 | GO:0006092 | main pathways of carbohydrate metabolism | 206 | 99 | 1.06 | 1.01E-09 |
| 35 | GO:0006260 | DNA replication | 282 | 108 | 1.05 | 3.74E-11 |
| 36 | GO:0043065 | positive regulation of apoptosis | 283 | 117 | 1.04 | 3.16E-11 |
| 37 | GO:0007266 | Rho protein signal transduction | 207 | 87 | 1.03 | 2.35E-11 |
| 38 | GO:0043066 | negative regulation of apoptosis | 284 | 115 | 1.03 | 5.32E-11 |
| 39 | GO:0006816 | calcium ion transport | 208 | 86 | 1.02 | 4.40E-11 |
| 40 | GO:0007067 | mitosis | 268 | 114 | 1.02 | 1.01E-10 |
| 41 | GO:0006917 | induction of apoptosis | 241 | 106 | 1.01 | 9.19E-11 |
| 42 | GO:0045934 | negative regulation of nucleobase, nucleoside, nucleotide and nucleic acid metabolism | 179 | 70 | 1.00 | 9.91E-05 |
| 43 | GO:0008284 | positive regulation of cell proliferation | 209 | 94 | 1.00 | 5.06E-11 |
| 44 | GO:0016481 | negative regulation of transcription | 274 | 109 | 0.99 | 2.41E-11 |
| 45 | GO:0000087 | M phase of mitotic cell cycle | 239 | 104 | 0.98 | 5.62E-11 |
| 46 | GO:0000165 | MAPKKK cascade | 214 | 85 | 0.97 | 9.36E-05 |
| 47 | GO:0006814 | sodium ion transport | 198 | 105 | 0.97 | 2.53E-11 |
| 48 | GO:0045045 | secretory pathway | 288 | 115 | 0.96 | 3.26E-11 |

|  | **GO ID** | **GO Class** | **Probes in class** | **Genes in class** | **Raw score** | **FDR** |  |
| --- | --- | --- | --- | --- | --- | --- | --- |
| 1 | GO:0016072 | rRNA metabolism | 149 | 75 | 0.83 | 5.06E-10 | **C18:1** |
| 2 | GO:0016070 | RNA metabolism | 187 | 75 | 0.81 | 1.44E-10 |
| 3 | GO:0007266 | Rho protein signal transduction | 207 | 87 | 0.81 | 1.26E-10 |
| 4 | GO:0000087 | M phase of mitotic cell cycle | 239 | 104 | 0.79 | 2.53E-10 |
| 5 | GO:0007067 | mitosis | 268 | 114 | 0.78 | 3.37E-10 |
| 6 | GO:0007409 | axonogenesis | 275 | 112 | 0.77 | 1.01E-09 |
| 7 | GO:0043066 | negative regulation of apoptosis | 284 | 115 | 0.75 | 2.02E-10 |
| 8 | GO:0007243 | protein kinase cascade | 278 | 109 | 0.73 | 1.69E-10 |

|  | **GO ID** | **GO Class** | **Probes in class** | **Genes in class** | **Raw score** | **FDR** |  |
| --- | --- | --- | --- | --- | --- | --- | --- |
| 1 | GO:0009725 | response to hormone stimulus | 35 | 13 | 1.81 | 5.06E-10 | **C18:2** |
| 2 | GO:0006637 | acyl-CoA metabolism | 24 | 14 | 1.78 | 1.69E-10 |
| 3 | GO:0006333 | chromatin assembly or disassembly | 110 | 37 | 1.16 | 1.12E-10 |
| 4 | GO:0035023 | regulation of Rho protein signal transduction | 145 | 63 | 0.96 | 1.44E-10 |
| 5 | GO:0007266 | Rho protein signal transduction | 207 | 87 | 0.94 | 9.19E-11 |
| 6 | GO:0008284 | positive regulation of cell proliferation | 209 | 94 | 0.93 | 2.02E-10 |
| 7 | GO:0051056 | regulation of small GTPase mediated signal transduction | 195 | 86 | 0.91 | 1.01E-09 |
| 8 | GO:0019752 | carboxylic acid metabolism | 215 | 115 | 0.91 | 2.53E-10 |
| 9 | GO:0001558 | regulation of cell growth | 228 | 94 | 0.85 | 1.01E-10 |
| 10 | GO:0043067 | regulation of programmed cell death | 238 | 107 | 0.82 | 3.37E-10 |
| 11 | GO:0007167 | enzyme linked receptor protein signaling pathway | 305 | 115 | 0.82 | 1.26E-10 |

|  | **GO ID** | **GO Class** | **Probes in class** | **Genes in class** | **Raw score** | **FDR** |  |
| --- | --- | --- | --- | --- | --- | --- | --- |
| 1 | GO:0006637 | acyl-CoA metabolism | 24 | 14 | 2.48 | 1.26E-10 | **C18:3** |
| 2 | GO:0006732 | coenzyme metabolism | 63 | 35 | 1.45 | 8.43E-11 |
| 3 | GO:0019752 | carboxylic acid metabolism | 215 | 115 | 1.11 | 2.02E-10 |
| 4 | GO:0008203 | cholesterol metabolism | 122 | 55 | 1.02 | 3.37E-10 |
| 5 | GO:0006445 | regulation of translation | 133 | 48 | 0.99 | 1.12E-10 |
| 6 | GO:0006986 | response to unfolded protein | 138 | 64 | 0.94 | 1.01E-09 |
| 7 | GO:0006260 | DNA replication | 282 | 108 | 0.89 | 1.01E-10 |
| 8 | GO:0006928 | cell motility | 225 | 84 | 0.88 | 7.78E-11 |
| 9 | GO:0051242 | positive regulation of cellular physiological process | 201 | 91 | 0.83 | 2.53E-10 |
| 10 | GO:0043066 | negative regulation of apoptosis | 284 | 115 | 0.82 | 1.69E-10 |
| 11 | GO:0043065 | positive regulation of apoptosis | 283 | 117 | 0.81 | 9.19E-11 |
| 12 | GO:0006917 | induction of apoptosis | 241 | 106 | 0.79 | 5.06E-10 |
| 13 | GO:0007243 | protein kinase cascade | 278 | 109 | 0.78 | 1.44E-10 |

|  | **GO ID** | **GO Class** | **Probes in class** | **Genes in class** | **Raw score** | **FDR** |  |
| --- | --- | --- | --- | --- | --- | --- | --- |
| 1 | GO:0006637 | acyl-CoA metabolism | 24 | 14 | 2.20 | 1.01E-10 | **C20:5** |
| 2 | GO:0006732 | coenzyme metabolism | 63 | 35 | 1.33 | 8.43E-11 |
| 3 | GO:0019752 | carboxylic acid metabolism | 215 | 115 | 0.90 | 1.44E-10 |
| 4 | GO:0006986 | response to unfolded protein | 138 | 64 | 0.86 | 5.06E-10 |
| 5 | GO:0048514 | blood vessel morphogenesis | 236 | 98 | 0.83 | 1.69E-10 |
| 6 | GO:0009607 | response to biotic stimulus | 145 | 73 | 0.82 | 9.19E-11 |
| 7 | GO:0007243 | protein kinase cascade | 278 | 109 | 0.82 | 1.12E-10 |
| 8 | GO:0007067 | mitosis | 268 | 114 | 0.81 | 2.02E-10 |
| 9 | GO:0001525 | angiogenesis | 209 | 92 | 0.81 | 7.78E-11 |
| 10 | GO:0016568 | chromatin modification | 284 | 107 | 0.80 | 3.37E-10 |
| 11 | GO:0000087 | M phase of mitotic cell cycle | 239 | 104 | 0.78 | 1.26E-10 |
| 12 | GO:0030036 | actin cytoskeleton organization and biogenesis | 299 | 112 | 0.77 | 1.01E-09 |
| 13 | GO:0006325 | establishment and/or maintenance of chromatin architecture | 299 | 112 | 0.76 | 2.53E-10 |

|  | **GO ID** | **GO Class** | **Probes in class** | **Genes in class** | **Raw score** | **FDR** |  |
| --- | --- | --- | --- | --- | --- | --- | --- |
| 1 | GO:0006637 | acyl-CoA metabolism | 24 | 14 | 2.77 | 9.19E-11 | **C22:6** |
| 2 | GO:0006732 | coenzyme metabolism | 63 | 35 | 1.74 | 5.32E-11 |
| 3 | GO:0009408 | response to heat | 51 | 25 | 1.50 | 5.06E-10 |
| 4 | GO:0019395 | fatty acid oxidation | 38 | 21 | 1.50 | 7.22E-11 |
| 5 | GO:0019752 | carboxylic acid metabolism | 215 | 115 | 1.25 | 1.26E-10 |
| 6 | GO:0006986 | response to unfolded protein | 138 | 64 | 1.23 | 2.02E-10 |
| 7 | GO:0051789 | response to protein stimulus | 123 | 56 | 1.21 | 1.01E-09 |
| 8 | GO:0009266 | response to temperature stimulus | 68 | 38 | 1.16 | 1.44E-10 |
| 9 | GO:0009607 | response to biotic stimulus | 145 | 73 | 1.15 | 5.95E-11 |
| 10 | GO:0016070 | RNA metabolism | 187 | 75 | 0.97 | 7.78E-11 |
| 11 | GO:0012502 | induction of programmed cell death | 182 | 81 | 0.95 | 6.74E-11 |
| 12 | GO:0045045 | secretory pathway | 288 | 115 | 0.94 | 6.32E-11 |
| 13 | GO:0043066 | negative regulation of apoptosis | 284 | 115 | 0.93 | 1.12E-10 |
| 14 | GO:0008610 | lipid biosynthesis | 230 | 108 | 0.93 | 2.53E-10 |
| 15 | GO:0030036 | actin cytoskeleton organization and biogenesis | 299 | 112 | 0.91 | 3.37E-10 |
| 16 | GO:0007243 | protein kinase cascade | 278 | 109 | 0.91 | 1.01E-10 |
| 17 | GO:0043065 | positive regulation of apoptosis | 283 | 117 | 0.90 | 5.62E-11 |
| 18 | GO:0007067 | mitosis | 268 | 114 | 0.88 | 1.69E-10 |
| 19 | GO:0006260 | DNA replication | 282 | 108 | 0.87 | 8.43E-11 |
