# Supplementary material for: Effect of Synthetic Dietary Triglycerides: A Novel Research Paradigm for Nutrigenomics
Source: PLoS One. 2008 Feb 27;3(2):e1681. doi: 10.1371/journal.pone.0001681 (PMC2244803; doi:10.1371/journal.pone.0001681)
Supplement: Table S4 — Overlap in overrepresented Gene Ontology classes between dietary unsaturated fatty acids and fenofibrate and WY14643 based on analysis with Functional Class Score method, FDR<0.0001. (0.04 MB DOC) [file pone.0001681.s007.doc]

| **Treatment** | **No. over-represented GO classes** | **No. GO classes overlapping with WY14643** | **% GO classes overlapping with WY14643** | **GO classes not in WY14643** |
| --- | --- | --- | --- | --- |
| WY14643 | 62 |  |  |  |
| fenofibrate | 48 | 40 | 83.3% | GO:0006084, acetyl-CoA metabolism;  GO:0006519, amino acid and derivative metabolism;  GO:0006695, cholesterol biosynthesis;  GO:0006816, calcium ion transport;  GO:0009308, amine metabolism;  GO:0009725, response to hormone stimulus;  GO:0016126, sterol biosynthesis;  GO:0043283, biopolymer metabolism |
| C18:1 | 8 | 5 | 62.5% | GO:0007409, axonogenesis;  GO:0016070, RNA metabolism;  GO:0016072, rRNA metabolism |
| C18:2 | 11 | 9 | 81.8% | GO:0007167, enzyme linked receptor protein signaling pathway;  GO:0009725, response to hormone stimulus |
| C18:3 | 13 | 11 | 84.6% | GO:0006445, regulation of translation;  GO:0006928, cell motility |
| C20:5 | 13 | 13 | 100% | - |
| C22:6 | 19 | 18 | 94.7% | GO:0016070, RNA metabolism |
